# Context Matters: How Decontextualization Influences Public Perception and Conservation Attitudes Toward Barbary Macaques in Algeria

**DOI:** 10.3390/ani15223319

**Published:** 2025-11-17

**Authors:** Imane Razkallah, Sadek Atoussi, Thais Queiroz Morcatty, Rabah Zebsa, Cédric Sueur, Anne-Isola Nekaris

**Affiliations:** 1 Centre de Recherche en Environnement, Université Badji Mokhtar, Annaba 23000, Algeria; imenrazkallah@gmail.com; 2 Laboratoire de Recherche Biologie, Eau et Environnement, Université 8 Mai 1945, Guelma 24000, Algeria; zebsarabah@gmail.com; 3 Oxford Wildlife Trade Research Group, Oxford OX4 3AY, UK; thais.queiroz.morcatty-2018@brookes.ac.uk (T.Q.M.);; 4 School of Social Sciences, Oxford Brookes University, Headington Road, Oxford OX3 0BP, UK; 5 Université de Strasbourg, Centre Nationale de la Recherche Scientifique CNRS, Institut Pluridisciplinaire Hubert Curien IPHC UMR 7178, 67000 Strasbourg, France; cedric.sueur@iphc.cnrs.fr; 6 Anthropo-Lab ETHICS EA7446, Lille Catholic University, 59800 Lille, France; 7 Institut Universitaire de France, Saint-Michel 103, 75005 Paris, France

**Keywords:** content analysis, social media, wildlife, conservation attitudes, *Macaca sylvanus*

## Abstract

The decontextualization (the portrayal of wildlife removed from their natural ecological context through social media), can distort the public perception of these animals and harm conservation efforts. This paper presents an exploratory case study based on two highly visible Facebook videos. To explore this, we analyzed Facebook comments (*n* = 720) and emoji-based reactions (*n* = 23,024) regarding Barbary macaques (*Macaca sylvanus*) in two contexts: entertainment (macaque dressed in sports attire during political protests) and natural habitat (macaque being fed soda by tourists in its forest environment). This is the first study to examine how social media context influences public perception of Barbary macaque conservation status and welfare through analysis of viewer engagement on viral videos. The results indicated that videos depicting macaques in their natural habitat elicited significantly more positive conservation sentiments (68.4% of comments) compared to entertainment contexts (6.04% of comments). Conversely, the entertainment video generated predominantly negative conservation sentiments (54.95% of comments), with viewers expressing amusement rather than concern for species protection. Videos showing macaques in natural settings, particularly when depicting problematic feeding behaviors, prompted more critical engagement and awareness of conservation issues. This pattern suggests that anthropomorphized contexts may obscure recognition of species threats and normalize inappropriate human–wildlife interactions. Given the small dataset, these findings should be interpreted cautiously and as illustrative rather than generalizable. These findings lend preliminary support to the animal decontextualization hypothesis and underscore the importance of context in shaping public perceptions of wildlife and conservation priorities.

## Simple Summary

Wild animals are often shown on social media in ways that do not reflect their real lives, which can change how people think about them and even harm conservation. In this study, we looked at how people reacted to Barbary macaques, a threatened primate species, in two Facebook videos that each received over 500,000 views. We examined both written comments (720 in total) and emoji reactions (over 23,000) to videos showing macaques either in entertainment settings or in their natural habitat. We found that when macaques were shown in entertainment contexts, people were less likely to express concern for their conservation. However, when they were shown in the wild, especially when being fed by people, viewers were more critical and showed more negative reactions. These findings suggest that when animals are presented in human-like or playful ways, their real struggles and threats may be overlooked. This study highlights the importance of how animals are portrayed online, as it shapes public attitudes toward protecting them. Raising awareness of these hidden effects can help guide better communication strategies for conservation and ensure that social media supports, rather than harms, efforts to protect wildlife.

## 1. Introduction

In 2022, Internet usage reached 83.8%, with over 5.47 billion users worldwide [1]. The World Wide Web has long been used to support social connections, and Web 2.0 has accelerated the adoption of social media platforms. These systems allow users to collaborate on the creation of web content and to organize, index, edit, and comment on it. In recent years, social media has been used as a tool by researchers to study public perceptions of animals, inform conservation decisions, and monitor the online wildlife trade [2,3,4,5,6].

The rise of featuring non-human animals in the media has spurred the development of critical animal media studies and introduced the term ‘animal disneyfication.’ Coined to describe the perception of animals through stereotypical representations rather than real knowledge [7,8], ‘animal disneyfication’ adds a layer of distortion to public perceptions of wildlife through decontextualization, which refers to the portrayal of animals removed from their natural ecological context, such as depicting wild animals in human settings or engaging in human-like behaviors. Notably, research by Quarles et al. [9] and Ross et al. [10] emphasize that the dissemination of such material not only amplifies viewer reactions but also significantly influences the perception of primates as suitable pets, particularly when depicted in close proximity to humans. This effect further reinforces the notion of their non-threatened status in the wild. Conversely, other studies underscore the influence of such material on consumer demand for live wildlife and their derivatives (such as bushmeat, traditional medicine ingredients, and body parts), resulting in negative environmental implications [4,7,11,12,13,14,15,16,17]. This underscores the complex interplay between online content, public perception, and the environmental consequences associated with wildlife interactions.

The Barbary macaque (*Macaca sylvanus*) is the northernmost African non-human primate. Its current distribution ranges from a latitude of around 31°15′ N to 36°45′ N and from a longitude of around 7°45′ W to 5°35′ E, where it is found in three countries, Algeria, Morocco, and the British territory of Gibraltar in southern Spain [18]. The IUCN RED LIST (International Union for Conservation of Nature) has listed the species as endangered due to a substantial drop in their numbers in Algeria and Morocco in recent decades [19]. This decline saw the population decrease from an estimated 21,000 individuals in the 1970s to an estimated range of 5000 to 6000 individuals by 2009 [20]. More recent surveys in 2023 documented new occurrence data in Skikda and Jijel, estimating the Algerian population at approximately 9000 individuals, suggesting potential population recovery in some areas despite continued threats [21]. The primary factors contributing to this decline are habitat fragmentation resulting from recurrent forest fires in the region and commercial exploitation [3,18].

The presence of the Barbary macaque in certain regions of Algeria boosted tourism, with the forests occupied by macaque troops in the provinces of Jijel and Bejaia emerging as significant tourist attractions. Outside their home range, illegally wild-caught individuals are exhibited for photo props in the majority of touristic sites in Algeria, mainly in places frequented by holidaymakers—both locals and foreigners. The ability to be in close proximity to these wild animals can have distorting effects on our perceptions of their protection status, in addition to posing a risk of injury to the animals and visitors [7,22,23,24].

The close proximity of these monkeys to humans naturally leads to the sharing of content depicting human interactions with them on social media. In this study, we examine two such incidents where videos of Barbary macaques went viral, each accumulating over half a million views. Our goal was to analyze textual comments and emoji reactions on two Facebook videos to understand how users perceive the conservation status of this species—and, more broadly, conservation issues—depending on the context in which the content is presented. The first video showcased a Barbary macaque in an urban setting amid a crowd engaged in political protests, while the second depicted a macaque in its natural habitat being fed a soda drink by tourists.

To assess the nature of comments posted by viewers for each video type, we analyzed the proportion of comments and reactions categorized as either conservation-negative or conservation-positive. To gain deeper insights, we analyzed the proportion of comments that align with Kellert’s classification of fundamental attitudes toward animals [24]. This framework helps identify whether viewers perceive animals primarily as esthetically pleasing (esthetic), as companions (dominionistic), as part of the natural world (naturalistic, ecologistic, or scientific), or through other lenses such as moralistic or humanistic perspectives—offering a more nuanced understanding of how people react to viral animal videos. These attitudes, even if they were defined a few decades ago, are still relevant today and can be adapted to describe opinions held toward animals in different cultural contexts [15,25]. Additionally, this typology was previously used to assess the public perception of wildlife on social media [15,26], and to evaluate the impact on viewers when conservation organizations use animal images [5].

Based on the animal decontextualization hypothesis and previous research on primate portrayal in media [9,10,26,27,28,29,30,31], we predicted that the following: (1) the entertainment context video would elicit fewer conservation-positive comments and more amusement-oriented responses than the natural habitat video; (2) anthropomorphized presentation would reduce expressions of concern about species threats; (3) even problematic human–wildlife interactions (tourist feeding) shown in natural settings would generate more critical conservation awareness than entertainment contexts. Understanding these perceptions is essential for informing environmental sustainability strategies, as digital content influences public awareness, policy support, and consumer behavior. By identifying how social media representations can either hinder or promote conservation goals, this study contributes to broader discussions on the role of digital platforms in advancing biodiversity conservation and sustainable human–wildlife interactions. However, this analysis is based on a very limited sample of only two videos. It should be considered an exploratory case study rather than a generalizable assessment of public perception.

## 2. Materials and Methods

### 2.1. Video Selection and Data Collection

We compared two videos, both receiving 500,000 views at the time of data collection. The first video was accidentally found by the first author and was posted on 15 March 2019 on the Facebook page of fans of ‘Mouloudia club Alger’ (accessed on 9 April 2020) soccer club (hereafter entertainment setting video). In this video, a Barbary macaque was dressed in the national football team attire, carried on the shoulders of a protester, and mimicking as if the animal were the spokesperson. All of this staging took place in an anthropomorphized environment during demonstrations in Algeria aimed at reforming the political system.

We subsequently searched for other videos on YouTube and Facebook using the keyword “singe magot”, which means Barbary macaque in French, having received at least 500,000 views and a significant number of comments that would allow us comparisons with the first video selected. We found only one other video meeting these criteria. The second video selected depicted a Barbary macaque in its native habitat, which had become a touristic stop due to the expansion of the road network and the growth of the tourism industry, with visitors providing the monkey with soda drinks. The video was posted on ‘La beauté kabyle’ Facebook page on 17 July 2020 and accessed on 16 March 2021 (hereafter natural environment video). We collected information on the number of views, number of shares, number and nature of emoji’s, and analyzed both textual and emoji comments. As the number of textual comments was relatively low, and the majority of them were short, all the comments were read and coded. Comments with different interpretations were read a second time to limit subjectivity. For comments written in Berber, the authors asked help from a native Berber speaker.

### 2.2. Data Analysis

A total of 1055 textual comments were collected. After excluding comments that were tags, replies to tags and comments made as GIFs (graphic interchange formats), we obtained a dataset of 720 comments, as well as the Facebook reactions for each video. From a conservation perspective, comments expressing a desire to own a monkey as a pet (e.g., statements such as “I want one,” inquiries about where to buy one, or how to care for one) or remarks about the animal being cute or the video being funny were categorized as negative or detrimental to conservation efforts. In contrast, comments were classified as conservation-positive when they expressed disagreement with the video’s content, such as highlighting the species’ protected status or emphasizing that wild animals should not be fed or kept in human-altered environments. Accordingly, for emojis, we considered that the LIKE, Haha (LOL), and LOVE emojis expressed negative sentiments, while sad and angry faces expressed positive ones. We acknowledge that this classification is based on interpretive assumptions about user intent. “Like,” “Haha,” and “Love” reactions were classified as potentially detrimental to conservation not because they are inherently negative, but because they suggest entertainment-oriented engagement rather than conservation concern. This classification aligns with research showing that emoji meanings are context-dependent and that engagement-focused reactions may normalize problematic human–wildlife interactions. Then, we descriptively analyzed the dataset based on comment content using an adapted version of Kellert’s typology of attitudes toward animals [24].

We combined ecological and naturalistic comments into a single category in our analysis, as both concepts involve an appreciation of nature. The subtle difference between the two, where the first is more focused on a love for the natural world, and the second is centered on the protection of the environment and ecosystems may not be distinguishable through short comments [15,25]. We also added new categories labeled as other to include comments that did not fit into any of Kellert’s categories (Table 1), such as political comments. The latter were related to the political situation of the country in 2019, and to the situation in which the animal was filmed. Comments unrelated to the video content, or those whose meaning was unclear were also categorized as “other”. We could not reliably determine commenter demographics such as age, gender, or nationality from available data. However, we used comment language as a proxy for potential cultural background.

For the statistical analysis, we utilized Pearson’s chi-squared test (to examine differences in the proportions of Facebook comments and reactions across sentiment categories (positive, negative, and neutral) and followed by the Cramér’s V effect size using rcompanion version 2.5/0 package. Additionally, we applied the same test to assess the distribution of comments across each category of Kellert’s typology. To enhance the interpretability of the Chi-square results, we employed the corrplot function from the R-package corrplot version 0.2-0 to visualize Pearson residuals. These analyses were conducted using R software (version 4.0.4), and statistical significance was determined at *p* < 0.001.

### 2.3. Ethical and Legal Consideration

Social media data are a powerful tool for understanding human–wildlife interactions, and as with any research involving people, the use of social media data requires the highest standards of confidentiality and data protection [27]. In order to minimize any potential negative impact on individuals, data were collected in compliance with the web site terms of service, anonymized, and no third parties were involved in the data collection and storage stages. The procedure was reviewed and approved by the scientific committee of the research laboratory “Biologie, Eau et Environnement” at 8 May 1945 University. Guelma, Algeria.

## 3. Results

### 3.1. Positive vs. Negative Sentiments

Overall, the two videos showed a significant difference in the proportion of positive and negative responses toward the conservation of the animal (Chi-squared = 226.56, df = 2, *p* < 0.001; Cramér’ V = 0.56). For the video set in an entertainment context, 54.95% of the comments reflected negative conservation sentiments. In contrast, the video depicting the animals in their natural environment had the majority of the comments (68.4%) expressing positive conservation sentiments (Table 2).

### 3.2. Emoji-Based Reaction Analysis

**Figure 1:**
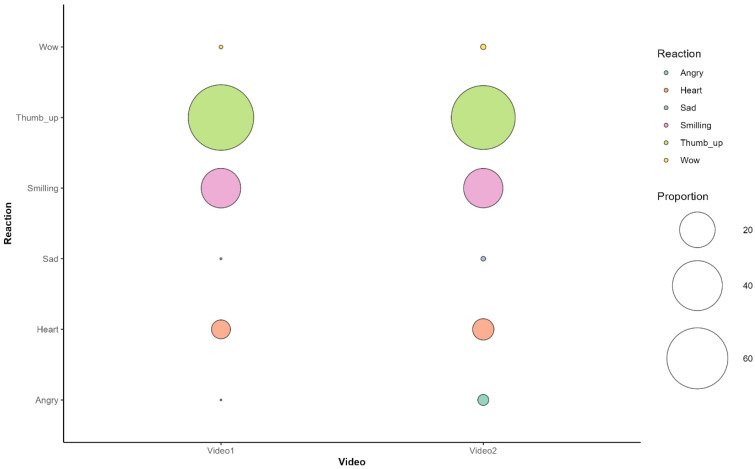
Distribution of emoji-based reactions across the two videos.

Emoji reactions far outnumbered textual comments, with 9227 and 13,797 emoji-based reactions for the entertainment and natural environment videos, respectively. A comparison between the two videos revealed a significant difference in emoji-based reactions (Chi-squared = 211.21, df = 5, *p* < 0.001; Cramér’ V = 0.096). In both videos, the most commonly used emojis were “Like,” “Haha” (LOL), and “Love” emoji’s typically associated with entertainment-oriented engagement. These three emoji types accounted for nearly 100% of the reactions in the entertainment setting video and 97.6% in the natural environment video. The “Sad” and “Angry” emojis were used less frequently, representing only 0.05% of reactions in the entertainment setting video and 2% in the natural environment video. The entertainment setting video was positively associated with the “Like” (thumbs up) emoji, with fewer of the other reactions than expected. In contrast, the natural environment video was more strongly associated with the “Angry” face reaction, though it also received “Sad,” “Love,” and, to a lesser extent, “Wow” reactions (Figure 1).

### 3.3. Adapted Kellert’s Categories

The entertainment setting video and the natural environment video differed significantly in the distribution of Kellert’s categories (Chi-squared = 227.15, df = 5, *p* < 0.001; Cramér’ V = 0.56). The primary differences emerged in comments reflecting ecologistic, naturalistic, or moralistic expressions. In the natural environment video, 68.4% of the comments fell into these three categories, compared to only 6.05% in the entertainment video (Table 3). Additionally, the entertainment video was more positively associated with other and funny comments, whereas the natural environment video was more closely linked to naturalistic and moralistic comments. The entertainment setting was significantly less associated with naturalistic and moralistic responses.

### 3.4. Language-Specific Patterns

Language distribution differed markedly between videos (Table 2). The entertainment video showed relatively balanced language use (Arabic: 46%, French: 29%, Arabic Latin characters: 23%), while the natural environment video was dominated by French comments (72%), with Arabic (10%) and Berber (10%) being much less represented. This pattern suggests potential audience composition differences, with the tourism video likely attracting more French-speaking viewers, whether international tourists or French-educated Algerians. Berber-language comments were much more prevalent in the natural environment video (56 vs. 4 comments), reflecting local community engagement with content from the Kabyle region. Analysis of Berber comments showed 24% conservation-positive sentiments, with particular emphasis on moralistic/humanistic categories. Due to limited sample sizes, we could not conduct robust statistical comparisons across language groups. However, descriptive patterns suggest that French is more used to express conservation-positive sentiments.

## 4. Discussion

It is clear today that social media has great potential for use in raising awareness and environmental education, given the growing audience it generates. However, several studies emphasize the need for caution when using and sharing materials (photos or videos) that feature wild animals on these platforms [28]. Indeed, Shaw et al. [5], in a study on the analysis of images used for biodiversity conservation awareness by NGOs, provide a set of criteria to be followed to avoid distorting the perception of the animals in question, such as the presence of humans which can lead to unintended effects. Likewise, several other studies exploring reactions to viewing videos featuring primates highlight these perception distortions [9,26], and all converge toward the conclusion that the context in which animals are shown determines perception and conditions reactions.

By comparing viewers’ reactions to two Facebook videos depicting a Barbary macaque in both anthropomorphized (entertainment) and natural environments (with tourist interaction), the present work constitutes the first study examining the influence of context in people’s perception of the Barbary macaque’s conservation status and welfare through the lens of social media engagement.

Our results demonstrate that animal depiction in non-natural environments may significantly disrupt the connection between fundamental environmental values and conservation attitudes [26,28]. Previous studies have shown the role of attitudes and perception in influencing the decisions made by biodiversity management agencies, as well as the relationship of such psychological dimensions with demographic variables.

Our results suggest that the way wildlife is shown in specific contexts can activate mutualism (a perspective viewing wildlife as deserving of rights and care) tends to be activated by natural settings, while dominance (a perspective viewing wildlife as resources for human benefit) tends to be activated by anthropomorphized settings. This is particularly relevant for mammals and non-human primates, where the anthropomorphic vision that underlies human preference for phylogenetically closer species may further complicate human–wildlife relationships [5,26,29]. Moreover, decontextualization (removing animals from their natural habitat) may lead to perception of wild species as abundant or domesticated rather than endangered [30,31]. This false sense of security about species well-being can reduce the perceived urgency for conservation actions and minimize awareness of human-induced threats such as habitat degradation and overexploitation [32].

Another critical consequence of decontextualization is the normalization of harmful human wildlife interactions such as feeding wild animals or using them as props for selfies. This shifts the focus from conservation concern to engagement driven content, where animals are treated as entertainment rather than sentient beings requiring protection [33]. Social media platforms, driven by algorithms that prioritize viral and visually appealing content, can inadvertently encourage these misperceptions and reinforce behaviors that are detrimental to conservation efforts. Our interpretation framework of emojis acknowledges their inherent polysemic and context-dependent meaning [34,35,36]. From this perspective, we argue that the dominance of superficially positive reactions (“Like,” “Haha,” “Love”) in both contexts, as well as the significant association between entertainment contexts and “Like” reactions, contrasted with natural contexts’ stronger association with “Angry” and “Sad” reactions, suggesting that viewing context influences both the type and potential meaning of emotional responses. We interpret “Like,” “Haha,” and “Love” reactions not as definitively negative conservation sentiments, but rather as indicators of entertainment-oriented engagement that may normalize problematic human–wildlife interactions and reflect emotional incongruence with conservation concerns. This pattern becomes particularly concerning when considering the role of social media in shaping public perceptions of wildlife [15]. The stark contrast in emoji distribution with 99.9% entertainment-oriented reactions in the anthropomorphized context versus 97.6% in the natural context provides contextual validation for our interpretive approach, suggesting that users respond differently to wildlife content based on its presentation.

Although direct comments expressing a desire to keep a monkey as a pet were absent, comments that described the animal as “cute” or the video as “funny” subtly introduce the notion of “cuteness.” This can alter perceptions of wild animals and potentially fuel demand in the illegal wildlife trade [11,16,26,31,33].

The situation in Morocco and Algeria, where Barbary macaques are frequently used as photo props at tourist sites, exemplifies how repeated exposure to anthropomorphized contexts can normalize dominionistic contexts [2,37]. Our findings suggest that such practices, combined with social media representations, may be creating a feedback loop that reinforces dominance at the expense of mutualism that support conservation efforts.

### Study Strengths, Limitations and Future Direction

This study provides the first examination of how social media context influences conservation attitudes toward Barbary macaques in Algeria. We analyzed authentic viral videos and combined multiple data streams textual comments, emoji reactions, and linguistic patterns across four languages, to capture real world dynamics. Our focus on an Algerian case contributes to underrepresented non-Western perspectives in wildlife social media research.

Despite being limited in our study to analyzing comments from only two videos and facing challenges in interpreting a large number of reactions, many of which were tags or ambiguous comments, our study also relies on inferred meanings of emoji-based reactions such as LIKE, Haha (LOL), and LOVE. While we have offered an interpretation of these reactions, we acknowledge that their intended meaning remains uncertain, as we were unable to validate our assumptions through direct user interviews. Future research would benefit from analyzing a larger sample of videos to better distinguish between different contextual elements that might influence value orientations. Expanding research to include multiple species would also help determine whether these patterns in public perception are unique to Barbary macaques or extend to other wildlife species. Furthermore, longitudinal studies could help understand how repeated exposure to different contexts might shape public perceptions of wildlife and conservation priorities over time. The stark difference in French-language comments between videos raises questions about whether language serves as a proxy for educational background or conservation awareness in Algeria. French is associated with higher education and access to scientific literature in the Algerian context. However, our sample size was insufficient to conduct rigorous language-stratified analysis, and we lack baseline data on whether French speakers in Algeria indeed demonstrate different conservation attitudes than Arabic or Berber speakers. Critically, baseline research is needed to understand language-specific patterns in conservation awareness within Algeria. Does language preference correlate with educational attainment, access to conservation information, or environmental attitudes? A structured questionnaire assessing how different segments of the Algerian population (by region, language, education level, urban vs. rural background) perceive biodiversity and conservation would be invaluable. Establishing these baseline relationships would enable future studies to account for or control language-associated confounds when analyzing social media responses to wildlife content.

## 5. Conclusions

This exploratory study provides initial insights into how social media context shapes public response to endangered wildlife content, specifically within the Algerian cultural and political context. Our analysis reveals a potential paradox: while social media offers unprecedented reach for conservation messaging, the medium’s preference for engaging, anthropomorphized content may inadvertently undermine conservation goals. Despite the mentioned limitations, our case study establishes a methodological foundation for broader research into social media’s role in shaping conservation attitudes across species and cultural contexts. These findings highlight the need for developing evidence-based guidelines for wildlife representation that prioritize conservation messaging over viral engagement metrics and underscore the critical responsibility of social media platforms to promote responsible wildlife content that supports rather than undermines global conservation efforts.

## Figures and Tables

**Table 1:** Kellert’s typology categories used in this analysis to assess online opinions toward macaque, with examples of original comments and English translation.

Kellert Category	Definition of Attitudes According to Kellert 1980 [25]	Original Comment	English Translation
Naturalistic and ecologistic	Exhibition of interest in and affection for wildlife and the outdoors, while also possessing an ecologistic mindset, demonstrating a primary concern for the environment as a system and the interrelationships between wildlife species and their natural habitats.	“Laisser les animaux vivre à l’état naturel et sauvage” “Respecter les animaux svp Me singe doit être dans son environnement”	“Let the animals live in their natural and wild state” “Please respect the animals. This monkey should be in its natural environment”
Moralistic/humanistic	Primary concern for the right and wrong treatment of animals, with strong opposition to exploitation of and cruelty toward animals.	“L’ignorance. c’est pas amusant mais ça peut le tuer” “Il fait mal au cœur ce singe qui ne sait pas ce qui lui arrive, ni pourquoi il est tiraillé dans tous les sens” “Chadi n’a pas fait de mal a personnes”	“Ignorance. It’s no fun, but it can kill him” “It breaks the heart to see this monkey, who doesn’t know what is happening to him or why he is being pulled in all directions” “The monkey did not harm anyone”
Scientistic	Primary interest in the physical attributes and biological functioning of animals.	“Non c pas drôle … les singes magot est une rasse rare en algérie il faut pas les donnés à manger et surtout les boissons sucré Ils tombes malades du diabète et des cancer”	“No it’s not funny … the Barbary monkey is rare in Algeria we must not give them to eat especially sugary drinks They fall sick with diabetes and cancer”
Funny		“hhhh j’en peux plus”	“hhhh I can’t take it anymore”
Political	Comments related to the political context in Algeria and popular protests during which the video was filmed.	“على الأقل شادي ماشي سراق” “شادي اشرف من عصابة الحكومة”	“At least the monkey is not corrupt” “The monkey is more respectable than the members of the government”
Others		“Tags and other incomprehensible comments”	“Tags and other incomprehensible comments”

**Table 2:** Comments classification according to languages, emoji types and sentiment categories (numbers and percentage).

	Entertainment Setting Video	Natural Environment Video
Comments language		
Arabic	84	54
Arabic Latin characters	41	40
French	53	388
Berber	4	56
Total	182	538
Emoji types		
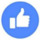	6.4 K (69.36%)	9.1 K (65.95%)
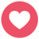	50 (5.5%)	971 (7.03%)
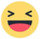	2.3 K (24.93%)	3.4 K (24.64%)
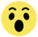	14 (0.15%)	49 (0.35%)
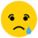	3 (0.03%)	36 (0.26%)
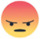	2 (0.02%)	241 (1.74%)
Total	9227 (100%)	13,797 (100%)
Sentiment categories		
Positive	11 (6.04%)	368 (68.4%)
Negative	100 (54.95%)	69 (12.82%)
Other	71 (39.01%)	101 (18.77%)
Total	182 (100%)	538 (100%)

**Table 3:** Comments classification according to Kellerts’ categories and others (numbers and percentage).

Comments	Naturalistic Ecologistic	Moralistic/Humanistic	Scientific	Esthetic	Funny	Other
Entertainment setting video	2 (1.1%)	8 (4.4%)	1 (0.55%)	3 (1.65%)	97 (53.29%)	71 (39.01%)
Natural environment video	207 (38.47%)	138 (25.65%)	23 (4.27%)	1 (0.18%)	69 (12.82%)	100 (18.58%)

## Data Availability

The datasets used and/or analyzed during the current study are available from the corresponding author on reasonable request.
